# Building a panel data set on fuel stations located in the Spanish regional areas of Madrid and Barcelona

**DOI:** 10.1016/j.dib.2016.01.063

**Published:** 2016-02-06

**Authors:** Jacint Balaguer, Jordi Ripollés

**Affiliations:** a Department of Economics and Instituto de Economía Internacional, Universitat Jaume I, Spain; b Department of Business Economics, Universitat de les Illes Balears, Spain

**Keywords:** Fuel stations, Fuel prices

## Abstract

The data described in this article were collected daily over the period June 10, 2010, to November 25, 2012, from the website of the Spanish Ministry of Industry, Energy and Tourism. The database includes information about fuel stations regarding to their prices (both gross and net of taxes), brand, location (latitude and longitude), and postal code in the Spanish provinces of Madrid and Barcelona. Moreover, obtaining the postal codes has allowed us to select those stations that are operating within the metropolitan areas of Madrid and Barcelona. By considering those fuel stations that uninterruptedly provided prices during the entire period, the data can be especially useful to explore the dynamics of prices in fuel markets. This is the case of Balaguer and Ripollés (2016), “Asymmetric fuel price responses under heterogeneity” [1], who, taking into account the presence of the potential heterogeneity of the behaviour of fuel stations, used this statistical information to perform an analysis on asymmetric fuel price responses.

**Specifications Table**

**Table:** 

Subject area	*Economics*
More specific subject area	*Energy Economics*
Type of data	*Spreadsheet*
How data was acquired	*Daily extractions from the website of the Spanish Ministry of Industry, Energy and Tourism* (http://geoportal.mityc.es/) *during the period June 10, 2010–November 25, 2012*
Data format	*Raw*
Experimental factors	*Extraction of information about fuel stations in the provinces of Madrid and Barcelona, with the consequent generation of 900 spreadsheets corresponding to each of the days of extraction. Data consolidation using MATLAB. Collection of postal codes of each service station by the Google Maps Geocoding API application. Selection of specific stations from the metropolitan areas of Madrid and Barcelona by using the Stata software.*
Experimental features	*The data include information about prices, brand and geospatial characteristics of fuel stations*
Data source location	*Provinces of Madrid and Barcelona* (*Spain*)
Data accessibility	*Data is with this article*

**Value of the data**

• The data allow researchers to investigate the effect of spatial competition on price behaviour.
• Showing the potential importance of the brand differentiation on price behaviour.
• Empirical analyses on geographical market integration in fuel markets.
• Analyses of cost pass-through in fuel markets with micro-data.
• Comparisons of pricing behaviour with other geographical and product markets.

## 1 Data

In this article we share two spreadsheets, which contain the following information for the period from June 10, 2010, to November 25, 2012:

Spreadsheet 1 Retail prices expressed in €/litre of diesel fuel (both gross and net of taxes), brands, locations (latitudes and longitudes) and postal codes of fuel stations in the Spanish provinces of Madrid and Barcelona.

Spreadsheet 2 Retail prices expressed in €/litre of diesel fuel (both gross and net of taxes), brands, locations (latitudes and longitudes) and postal codes of fuel stations in the Metropolitan areas of Madrid and Barcelona.

## 2 Experimental design, materials and methods

The primary source of our databases is the web application of the Spanish Ministry of Industry, Energy and Tourism called *GEOPORTAL* (www.geoportalgasolineras.es). This web application is based on the information provided directly by the fuel stations operating in Spain which, in accordance with Ministerial Order ITC/2308/2007, are required to submit current retail prices every Monday and whenever price changes are applied. Data offered online by the Spanish Ministry are available only in real time and the historical series are not provided. Thus, we have extracted daily, throughout the period from June 10, 2010, to November 25, 2012, statistical information about consumer prices (gross of taxes) on diesel fuel in the provinces of Madrid and Barcelona. Each of the nine hundred spreadsheets (*.*csv*) obtained for each province also includes the corresponding brands and locations (latitudes and longitudes) of the fuel stations. Extractions for each of the days were performed manually to avoid the possible consequences of eventual changes in the web application over the above-mentioned period.

We are interested in presenting the statistical information obtained in a useful panel database form that includes all the individuals in each province (*j*), (*N_j_*), that operate during any of (*T*) days considered. That is, we have transformed the 900 spreadsheets we had available into a single spreadsheet that includes a matrix ($N_{j}\mathrm{xT}$) for each province. By using the location variable to define each fuel station, we merged all the 900 daily files with the procedure “MainFile.m” programmed with MATLAB software. Moreover, we used the Google Maps Geocoding API application to obtain a postal code variable associated to each of the *N* fuel stations. This procedure was also programmed with MATLAB software and the code file was called “Google.m”. The MATLAB programming codes and final data for both provinces (labelled as “Spreadsheet 1”) are available in the Appendix B to this article. In the data provided we also included a column with the retail prices net of taxes for each fuel station (*i*) at each time (*t*), (*PN_it_*). In order to exclude taxes we applied the following equation:

(1) $${\mathrm{PN}}_{\mathrm{it}}=\frac{P_{\mathrm{it}}}{1+\mathrm{VAT}}-\left(\mathrm{SHT}+\mathrm{RSTCH}\left(j\right)\right)$$

where $P_{\mathrm{it}}$ represents final consumer prices, VAT represents the general Value Added Tax, SHT is the Special Hydrocarbons Tax, which is common to the Spanish regions of the peninsula, and RSTCH is the Retail Sales Tax on Certain Hydrocarbons. This latter tax includes a State section, which is common to the regions, and another section that depend of the region (*j*) and which, for our period, differs between Madrid and Barcelona (Cataluña). Information about taxes applied during the sample period can be obtained from the Spanish Ministry of Economy’s Tax Office. It has been summarised in Appendix A.

We also selected and provided the statistical information related to the fuel stations located in the metropolitan areas of Madrid and Barcelona, which are the two largest in Spain. This selection prevents the inclusion of fuel stations that are very far away from each other and, as in Barron et al. (2004) [2] or Lewis (2008) [3], it provides us with a simple way of taking into account the possible effects of spatial competition on price formation. There is no official definition of metropolitan area in Spain. Hence, for the metropolitan area of Madrid we used the definition from García Ballesteros and Sanz Berzal (2002) [4]. In the case of Barcelona we followed the territorial division of the General Territorial Plan of Catalonia, in accordance with Autonomic Law 1/1995 (Official Gazette of the *Generalitat* of Catalonia 2032).

The postal codes obtained from the Google Maps Geocoding API allowed us to select, by using Stata, those stations that are operating within the metropolitan areas of Madrid and Barcelona. The Stata programming codes for carrying out the selection of fuel stations, and the final data for both metropolitan areas (with retail prices gross and net of taxes) are available in the Appendix B to this article (“Stata codes” and “Spreadsheet 2”, respectively). These last data were particularly useful in the recent paper by Balaguer and Ripollés (2016) [1]. In that paper the authors also obtained the distance between each fuel station and their neighbouring competitor. This calculation can be computed directly through the “geosphere” package available in the R software application. Moreover, since a dynamic econometric model is applied, those fuel stations that did not provide prices throughout the entire period were excluded in the above-mentioned paper.

## Figures and Tables

**Table A1:** Value Added Tax (VAT) and Special Hydrocarbons Tax (SHT) on diesel fuel.

	VAT			SHT
In force from	January 1 2006	July 1 2010	September 1 2012	June 13 2009
Iberian Peninsula	16%	18%	21%	307


Author׳s own elaboration from information from the Spanish *Ministry of Economy׳s Tax Office*. Values for SHT are expressed in Euros per 1000 liters of fuel.

**Table A2:** Retail Sales Tax on Certain Hydrocarbons (RSTCH) on diesel fuel.

	State section	Regional section	
In force from	January 1 2002	January 1 2006	April 1 2012
Barcelona (Cataluña)	24	24	48
Madrid	24	17	17


Author׳s own elaboration from information from the Spanish *Ministry of Economy׳s Tax Office*. Values are expressed in Euros per 1000 liters of fuel.

## Appendix A

See Table A1, Table A2
